# A New Advancement in Germination Biotechnology of Purple Creole Corn: Bioactive Compounds and In Situ Enzyme Activity for Water-Soluble Extract and Pan Bread

**DOI:** 10.3390/metabo14010035

**Published:** 2024-01-04

**Authors:** Glauce Kelly Silva do Nascimento, Michelle Santos Silva, Irene Andressa, Mariane Bittencourt Fagundes, Raquel Guidetti Vendruscolo, Josimar Rodrigues Oliveira, Milene Teixeira Barcia, Vivian Machado Benassi, Nathália de Andrade Neves, Cristiane Teles Lima, Marcio Schmiele

**Affiliations:** 1 Institute of Science and Technology, Federal University of Jequitinhonha and Mucuri Valleys (UFVJM), Diamantina 39100-000, MG, Brazil; glauce.kelly@ufvjm.edu.br (G.K.S.d.N.); michelle.santos@ufvjm.edu.br (M.S.S.); vivian.benassi@ict.ufvjm.edu.br (V.M.B.); nathalia.neves@ict.ufvjm.edu.br (N.d.A.N.); cristiane.lima@ufvjm.edu.br (C.T.L.); marcio.sc@ict.ufvjm.edu.br (M.S.); 2 Department of Food Science and Technology, Federal University of Viçosa (UFV), Viçosa 36570-900, MG, Brazil; irene.andressa@ufv.br; 3 Department of Food Technology and Science, Federal University of Santa Maria (UFSM), Santa Maria 97105-900, RS, Brazil; milene.barcia@ufsm.br; 4 Institute of Agrarian Science, Federal University of Jequitinhonha and Mucuri Valleys (UFVJM), Diamantina 39100-000, MG, Brazil; josimar.oliveira@ufvjm.edu.br

**Keywords:** agrobiodiversity, bioprocess, breadmaking, phenolic compounds, technological aid

## Abstract

Germination is a simple and cost-effective technology that enhances the technological, sensory, and nutritional potential of grains, making them more attractive for use in the food industry. Germinating indigenous seeds is an alternative to increase noticeability and add value to these grains, which hold social and economic significance in the regions where they are cultivated, such as creole purple pericarp corn (PPCC) from the Couto Magalhães de Minas region in Brazil. This study aimed to optimize the germination parameters of time (24–96 h) and temperature (18–32 °C) for PPCC to produce water-soluble extracts and bread. Endogenous enzymes resulting from the germination process significantly enhanced (*p* < 0.10) the technological (total reducing sugars, total soluble solids, and soluble proteins) and biological properties (*γ*-aminobutyric acid, total soluble phenolic compounds, and antioxidant capacity) of the water-soluble extracts. The optimum point for obtaining the extracts was found to be at 85.3 h at 30.46 °C (with desirability of 90.42%), and this was statistically validated. The incorporation of germinated PPCC flours into bread was also promising (*p* < 0.10) and had a positive impact on the dough property (dough volume increase) and the final product, especially in terms of instrumental texture (springiness, cohesiveness, gumminess, chewiness, and resilience), resulting in a softer texture (lower firmness and hardness). The addition of PPCC flours did not alter instrumental color parameters, which may lead to greater consumer acceptance due to imperceptible differences in color to untrained individuals, with the optimized point at 96 h at 29.34 °C, with a desirability of 92.60%. Therefore, germinated PPCC shows promise for use as a base for obtaining water-soluble extracts and in bread as a replacement for commercial flour improvers, while also adding value to a raw material that is part of the local culture and agrobiodiversity.

## 1. Introduction

Cereals and cereal-based products are staple foods in both developed and developing countries, as well as being extensively used in animal feed. Grains serve as essential sources of carbohydrates and proteins, serving as the primary energy source for individuals [1]. Cereals are caryopses from plants belonging to the Gramineae family, and their morphological structure consists of the germ, endosperm, and pericarp [2]. In addition to their role as an energy reserve, cereals contain a metabolically active component responsible for the germination process.

Grain germination can be considered a simple and cost-effective method for enhancing the nutritional value of cereals and pseudocereals [3,4]. It is a bioprocessing technique that involves conditioning grains with high moisture levels (>40%) and mild temperatures (12–40 °C) to activate hydrolytic enzymes, such as amylases, glucanases, and proteases, which convert the endosperm into a source of energy and nitrogen. This results in the partial hydrolysis of macromolecules, releasing the nutrients and energy required for the embryo to develop sprouts and primary leaves, ultimately reaching its photosynthetic capacity. Germination is initiated through a single unit operation: the hydration of grains; this involves interactions between physical and hormonal factors, encompassing events from imbibition to radicle protrusion [5].

After water absorption by the seeds, appropriate conditions of temperature, light, and humidity trigger the transition of the grain from a state of dormancy to the beginning of active metabolism. This process is driven by the activation of multiple hydrolytic enzymes and hormones, such as gibberellin, which migrate from the embryo to the aleurone layer, facilitating the release of degradative enzymes for storage macromolecules. The loosening of the cell wall and an increase in oxygen consumption mark the onset of a lag phase, during which water content is stabilized, biochemical events occur, and RNA and proteins are synthesized. This process leads to the weakening of the endosperm, crucial for seed germination, creating favorable conditions for the rupture of the endosperm by the radicle. The post-germinative phase begins with an intensified demand for water due to the growth of the radicle, which elongates and rapidly absorbs water. Simultaneously, there is a pronounced mobilization of seed reserves, such as proteins, lipids, and polysaccharides [6].

From a dietary perspective, germination leads to the release or bioconversion of essential metabolites such as amino acids and bioactive compounds, enhancing nutrient density, bioavailability, and bioaccessibility. Structural modifications occur during the germination of cereal grains, improving their physicochemical and cooking characteristics. Germination also increases the availability of free amino acids, reducing sugars, and stimulates the accumulation of ***γ***-aminobutyric acid (GABA), minerals, dietary fiber (mainly soluble fiber), and phenolic compounds, in addition to increasing antioxidant activity [7,8]. Consequently, cereal germination has been actively utilized to obtain new food components to produce cereal-based products.

Phenolic compounds are secondary metabolites that naturally occur in fruits and vegetables, with the primary types being phenolic acids (hydroxybenzoic and hydroxycinnamic acids), flavonoids (notably anthocyanins), and non-flavonoids, such as tannins. The structure of phenolic compounds consists of aromatic rings associated with hydroxyl groups, giving these compounds properties that have attracted scientific interest due to their potential as alternatives to synthetic antioxidants in industrial applications, in addition to their health benefits [3]. In vitro studies have shown that phenolic compounds possess antimicrobial, antioxidant, antiviral, anti-inflammatory, and vasodilatory properties [9].

GABA is a non-protein amino acid with four carbons found in animals, plants, and other organisms. This compound is related to plant growth regulation and stress tolerance, responding rapidly to various abiotic disturbances [10]. As explained by Li et al. [11], this inhibitory neurotransmitter plays a role in plant development, especially in pollen tube elongation, root growth, fruit ripening, and seed germination. It is a molecule of interest due to its various health benefits, including the inhibition of cancer cell proliferation, stimulation of apoptosis, regulation of blood pressure and cholesterol levels, pain reduction, and anxiety relief [12].

In addition to the characteristics mentioned, endogenous enzymes activated during the germination process can serve technological functions in food production processes. Grain endogenous enzymes are widely used in food production processes, especially in the brewing and distilled alcoholic beverage industry, as an efficient, safe, and environmentally friendly processing alternative. Therefore, enzymatic bioprocessing methods are currently used to produce various foods, including dairy products, baked goods, beverages, oils and fats, and meat products [13]. In baking, the enzyme classes typically used fall into the categories of oxidoreductases and hydrolases, such as amylase, protease, hemicellulases, and lipases. The addition of amylases primarily aims to provide metabolizable sugars for yeast and delay bread aging. Proteases contribute to reducing the size of gluten polymers, making the dough easier to mix. Xylanase, in turn, acts on the partial hydrolysis of water-unextractable arabinoxylans, converting them into water-extractable arabinoxylans, facilitating dough hydration, improving stability, and enhancing bread structure. The action of lipases and esterases plays an important role in the lipid fraction, releasing free fatty acids, monoacylglycerols, and diacylglycerols, favoring increased bread-specific volume, crumb softness, and shelf-life extension [14,15].

While extensive research has been conducted on the germination processes of grains (cereals, legumes, and pulses), the exploration of creole varieties is limited due to the difficulty of obtaining raw materials, since cultivation occurs on a small scale and by local producers. However, the technological and scientific development of creole varieties can benefit family farming, regional development, innovation, and sustainability, especially in the face of various adverse conditions the world is exposed to, such as wars, hunger, malnutrition, and environmental conditions, like heavy rains, prolonged droughts, cyclones, tornadoes, uncontrolled fires, and more.

Therefore, the objective of this study was to evaluate the effects of germination’s time and temperature on the physicochemical, technological, and nutritional properties of purple pericarp landrace maize and the use of germinated flour to obtain water-soluble extracts as a technological adjunct in loaf bread production.

## 2. Materials and Methods

### 2.1. Materials

The seeds of purple pericarp creole corn (PPCC) (2020 harvest) were donated by the Creole Corn Project of the Institute of Agricultural Sciences at the Federal University of Vales Jequitinhonha and Mucuri (UFVJM). The corn was grown at the Rio Manso Experimental Farm in Couto Magalhães de Minas (Brazil), and the project was registered under the number A5C29C1 in the National System for Management of Genetic Heritage and Associated Traditional Knowledge (SisGen) of the Ministry of the Environment of the Federative Republic of Brazil. The seeds were manually selected and stored in polyethylene terephthalate bottles at room temperature and without light. The corn variety used in this study is under investigation in the Integrated Cereals and Lipids Laboratory (LICEL) by our research group and exhibited protein content of 10.29 ± 0.49% (N = 6.25), starch of 63.86 ± 4.55%, ash content of 1.28 ± 0.02%, lipids of 5.27 ± 0.42%, and dietary fibers of 19.30 ± 1.32% (on a dry basis) [4]. All chemicals used were of standard analytical grade with adequate purity for the applied analytical methodologies.

### 2.2. Methods

#### 2.2.1. Experimental Desing

The performance of germination’s time and temperature was studied using the Response Surface Methodology, based on a Central Composite Design (CCD), with two independent variables, where x_1_ represents germination time and x_2_ represents germination temperature (Table 1), following the mathematical model (Equation (1)) proposed by Rodrigues and Iemma [16]. The ranges for the time *x* temperature binomial were established based on a previous study conducted by the LICEL research group [4].

(1) $$\;Y=\;\beta_{0}\;+\beta_{1}x_{1}\;+\beta_{2}x_{2}\;+\beta_{11}x_{1}^{\;\;2}\;+\beta_{22}x_{2}^{\;\;2}\;+\beta_{12}x_{1}x_{2}\;+\;\varepsilon$$

where *Y* is the value of the dependent variable; *β*_0,_ *β*_i_, *β*_ii_, and *β*_ij_ are the regression coefficients of constant, linear, quadratic, and interaction, respectively; *x*_i_ and *x*_j_ are the encoded values for the independent variables; and *ε* is the experimental error.

#### 2.2.2. Germination Process

Firstly, PPCC seeds (150 g) were disinfected in 0.75 L of a 0.25% NaClO (*v*/*v*) solution for 30 min. The seeds were then rinsed three times with distilled water to remove residual chlorine and reach a neutral pH. Subsequently, they were soaked in distilled water at a ratio of 1:5 (*w*/*v*) for 24 h at room temperature (~20 °C). After soaking, the seeds were arranged in a polyethylene tray (0.045 m^2^) with hydrophilic cotton layers at the bottom and top. The soaked seeds were placed in the inner part, separated by an intermediate layer of paper (0.044 g·m^−2^). All layers were moistened with distilled water, and the trays were incubated in an RFE38 BOD germination chamber (Lucadema, São José do Rio Preto, Brazil) and kept in a dark environment. To maintain the relative humidity of the environment, a polyethylene tray with 3 L of distilled water was placed at the bottom of the germination chamber, and the samples were sprayed with distilled water every 12 h. Relative humidity was maintained in the range of 80–90% based on wet-bulb and dry-bulb temperatures, following the psychrometric chart.

Immediately after the germination tests, the length of the radicle was measured, seed vigor was determined, and the seeds were then dried in a TE-394/1 oven (Tecnal, Piracicaba, Brazil) with air renewal and forced air circulation (1 m·s^−1^) at 45 °C for 12 h. The dehydrated seeds were cooled to room temperature, and the radicles were manually removed. The grains were ground using a Multi Grãos disk grinder (Malta, Caxias do Sul, Brazil) to a particle size smaller than 350 µm. The germinated corn flours were stored in biaxially oriented polypropylene packaging and kept in a BFEI-200 refrigeration counter (Polar Refrigeração, Contagem, Brazil) at 4 °C in a dark environment until analysis.

#### 2.2.3. Radicle Length and Seed Vigor

The physiological quality of the seeds was evaluated following the methodology outlined by Andressa et al. [4]. To determine radicle length, ten randomly selected seeds were measured using a 150 mm professional optical caliper (Western, Suzhou, China), and the results were expressed in mm. For seed vigor, 50 seeds were chosen at random, and the number of seeds with a visible radicle was recorded. Seed vigor was calculated using Equation (2).

(2) $$Seed\;Vigor\;(\%)=\left(\frac{S_{g}}{S_{t}}\right)\cdot 100$$

where: *S_g_* is the number of seeds that showed radicle development, and *S_t_* is the total number of seeds in the sample.

#### 2.2.4. Analysis of Flours from Germinated and Non-Germinated Seeds

The flours from the control and germinated PPCC were analyzed regarding total soluble phenolic compounds (TSPCs) and GABA content. The flours obtained in Section 2.2.2 were ground using a TE-350 ball mill (Tecnal, Piracicaba, Brazil) to reduce the particle size to less than 150 µm.

##### Total Soluble Phenolic Compounds (TSPCs)

The extraction followed the methodology described by Lima et al. [7], with some modifications. A total of 200 mg (in duplicate) of corn flour was weighed and placed in 2000 μL Eppendorf tubes. Then, 1500 μL of the optimized extraction solution, consisting of 53% acetone and 47% acetic acid (*v*/*v*) [17], was added. The extraction was carried out exhaustively in five consecutive cycles. The tubes were placed in a CBU/100/3LDG low-frequency ultrasonic bath (Planatec, São Paulo, Brazil) (40 kHz/100 W) at room temperature (~20 °C) for 5 min, followed by centrifugation at 5000× *g* for 10 min at 20 °C. At the end of each cycle, the supernatants were collected in a volumetric flask, and the final volume was adjusted to 10 mL. The absorbance of phenolic components was determined using the Folin–Ciocalteu method (1:9 in distilled water—*v*/*v*), with 3 mL of distilled water and 1 mL of 15% Na_2_CO_3_ (*w*/*v*). The color reaction was conducted in the absence of light for 30 min. Absorbance readings (ten repetitions for each replicate) were taken at 750 nm using an Anthos 200 ZT microplate spectrophotometer (Zenyth, São Paulo, Brazil), using transparent 96-well microplates. A 7-point standard curve was constructed using gallic acid (0 to 300 mg·L^−1^; y = 0.0015x − 0.0159; r = 0.9946).

##### *γ*-Aminobutyric Acid (GABA)

The quantification of GABA content was carried out following the methodology described by Lima et al. [7], with some modifications. Initially, 600 mg of the sample (in triplicate) was weighed in Falcon tubes, and 4.5 mL of the extraction solution (75% ethanol—*v*/*v*) was added. Ultrasonic-assisted extraction was performed using a CBU/100/3LDG low-frequency ultrasonic bath (Planatec, São Paulo, Brazil) (40 kHz, 100 W) for 5 min at room temperature (~20 °C), followed by phase separation using a Fanem Baby centrifuge (Tecnal, Piracicaba, Brazil) at 1832× *g* for 10 min. After centrifugation, the supernatant was transferred to a 10 mL volumetric flask. The extraction process was repeated with the addition of 4.5 mL of the extraction solution and resuspension using an NA 3600 vortex mixer (Norte Científica, Araraquara, Brazil). Finally, the volume was adjusted to 10 mL with the extraction solution and filtered through a Unifil filter paper (9 cm in diameter, 80 g·m^−2^).

The extracted GABA was derivatized to dansyl-*γ*-aminobutyric acid using 100 µL of aliquot, 200 µL of dansyl chloride (10 mM in acetonitrile (ACN)), and 200 µL of 0.5 M sodium carbonate buffer (pH 10), with immersion in a low-frequency ultrasonic bath for 3 min, followed by heating at 65 °C in an SL-150 static water bath (Solab, Piracicaba, Brazil) for 25 min and subsequent centrifugation at 9600× *g* for 15 min at 4 °C in an SL-5GR centrifuge (Spinlab, Ribeirão Preto, Brazil). The quantification of GABA was performed using a 1260 Infinity High-Performance Liquid Chromatography (HPLC) system (Agilent Technologies, Santa Clara, CA, USA).

To quantify the GABA content in the samples, a standard of *γ*-aminobutyric acid (Merk, purity >99%) was used within a linear range of six points (0 to 184 mg·L^−1^; y = 29.245x + 30.702; r = 0.9994). The HPLC system consisted of a quaternary pump and a G1314B diode array detector (DAD). The mobile phase consisted of a sodium phosphate buffer (0.05 M, pH 7.2) and ACN with a flow rate of 1.0 mL·min^−1^. All mobile-phase solvents were of HPLC grade. Initially, the chromatographic separation column (Zorbax Eclipse C18; 4.6 × 100 mm, 3.5 µm) was eluted with a linear gradient, in which the amount of ACN was gradually increased 14–20% ACN (0–7 min), 20–22% ACN (7–10 min), and 22–25% ACN (10–15 min). Subsequently, ACN was maintained at 85% for 3 min, followed by a linear gradient in which ACN was reduced from 85 to 14% (25 to 26 min). The injection volume was 10 µL, and detection was performed at 254 nm. The results were expressed in mg of GABA per 100 g of sample, on a dry basis.

#### 2.2.5. Obtaining Water-Soluble Extracts

**Figure 1:**
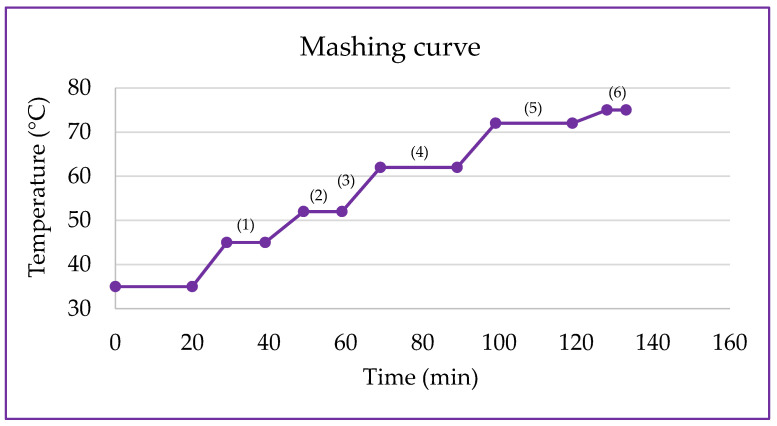
Mashing ramp for obtaining water-soluble extracts from germinated purple pericarp creole corn. (1) Optimal temperature for the action of hemicellulases (40–45 °C) and exopeptidases (40–50 °C). (2) Optimal temperature for the action of endopeptidases (50–60 °C). (3) The temperature range that includes the ramp favors the activity of dextrinases (55–60 °C). (4) Optimal temperature for the action of *β*-amylases (60–65 °C). (5) Optimal temperature for the action of *α*-amylases (70–75 °C). (6) Enzymatic inactivation (75 °C) [18].

The corn flours underwent the mashing process to obtain the water-soluble extracts in a water bath NT 232 (Novatecnica, Piracicaba, Brazil) according to the mashing ramp shown in Figure 1. A ratio of 1:6 (flour:distilled water—*w*/*v*) on a dry basis was utilized. After mashing, the samples were transferred to 50 mL Falcon tubes and subjected to phase separation in a Sorvall ST8 Centrifuge at 3200× *g* for 15 min (Thermo Fisher Scientific, Waltham, MA, USA). Subsequently, the extracts were filtered through hydrophilic cotton; the volume was adjusted to 100 mL in a volumetric flask and stored in rigid biaxially oriented polypropylene packaging under freezing conditions in a DFN41 freezer (Electrolux, Curitiba, Brazil) at −18 °C until the time of analysis.

#### 2.2.6. Characterization of Water-Soluble Extracts

The water-soluble extracts were characterized by their soluble protein content, soluble solids, and reducing sugars.

##### Soluble Protein

To digest the samples, 10 g of the extract was weighed and added to a protein digestion tube (in triplicate), followed by overnight drying in a TE-394/1 oven (Tecnal, Piracicaba, Brazil) with air circulation and renewal (1 m·s^−1^) at 105 °C to evaporate the water. The determination of the total nitrogen in the water-soluble extracts was carried out using the MicroKjeldahl method following AACCI method 46-13.01 (N = 6.25) (AACCI, 2010) [19], and the results were expressed as a percentage of soluble protein concerning the total protein present in the PPCC flour.

##### Soluble Solids

The content of soluble solids was determined following AACCI method 80-51.01 (AACCI, 2010) [19] through a direct reading on EEQ-9030 digital refractometer (Lorden, Porto Alegre, Brazil), previously calibrated with distilled water. The analysis was performed in triplicate, and the results were expressed in °Brix.

##### Reducing Sugars

The quantification of reducing sugars was determined by a colorimetric method using the 3,5-dinitrosalicylic acid (DNS) reagent, following the methodology proposed by Santos et al. [20], with modifications. The reaction was carried out in test tubes (in triplicate), using 30 µL of the water-soluble extract, 100 µL of distilled water, and 30 µL of DNS. The tubes were then heated to boiling in an SL-150 water bath (Solab, Piracicaba, Brazil) for 5 min. After cooling, 1000 µL of distilled water was added. To perform the quantification, 300 µL of the reaction mixture (in triplicate for each color reaction) was pipetted into a transparent 96-well microplate, and the samples were read at 540 nm using an Anthos 200 ZT microplate reader (Zenyth, São Paulo, Brazil). The concentrations (mg of glucose per mL of water-soluble extract) were calculated based on a linear equation (y = 0.464x; r = 0.9984), generated from a 5-point calibration curve with glucose concentrations of 0.2, 0.4, 0.6, 0.8, and 1.0 mg·mL^−1^.

#### 2.2.7. Numerical Optimization and Mathematical Models’ Validation for Germination Process and Water-Soluble Extracts

The optimization of the germination process was conducted following the methodology proposed by Derringer and Suich [21]. The independent variables were optimized within the studied range. Statistically significant dependent variables were set as maximum values, with each response assigned a level of importance (where 1 and 5 represented the lowest and highest levels of importance, respectively). The optimal point was tested in true triplicate to ensure the accuracy of the mathematical models’ predictions.

##### Antioxidant Capacity

The antioxidant capacity was evaluated in the water-soluble extract from the control and optimal point trials using the oxygen radical absorbance capacity (ORAC) method, as proposed by Dávalos [22], with modifications described by Camponogara et al. [23]. The extracts were obtained following the TSPC analysis. Initially, 20 µL of the extract or the standard 6-hydroxy-2,5,7,8-tetramethylchroman-2-carboxylic acid (Trolox) at concentrations ranging from 10.0 to 500 mM (8-point calibration curve, r = 0.9904) was pipetted. Then, 120 µL of 70 nM fluorescein was added to a 96-well black microplate, which was incubated at 37 °C for 15 min. Subsequently, 200 µL of 12 mM 2,2-azobis(2-methylpropionamidine) dihydrochloride (AAPH) was added. The reduction in fluorescence was measured on a SpectraMax^®^ Paradigm^®^ microplate reader (Molecular Devices, CA, USA) every min for 80 min, using wavelengths of 485 nm for excitation and 525 for emission. The plate was maintained at 37 °C throughout the assay, and the solutions were homogenized by orbital shaking for 5 s before each reading. The results were expressed in mg of Trolox per mL of the extract.

#### 2.2.8. Breadmaking Process

The standard bread formulation used for breadmaking included wheat flour (100%), palm fat (4%), salt (1.8%), instant yeast (1.2%), and potable water at 7 °C (58%) (on flour basis) [24]. For the CCD experiments and the control sample (non-germinated PPCC flour), wheat flour was replaced with 4% PPCC flour (*w*/*w*). The dough was prepared in an automatic Multi Pane bakery machine (Britânia, Joinville, Brazil), using the profile 1 program, which alternated two mixing periods with a dough resting time (1st mix for 10 min, rest for 20 min, and 2nd mix for 15 min). The dough was divided into 250 g portions, manually rounded and molded in an MPV35 molder (Venâncio, Venâncio Aires, Brazil), and transferred to pre-greased aluminum pans (9.5 × 4.5 × 24 cm—width, height, length, respectively). Fermentation and baking times were determined through preliminary tests. The doughs were fermented in a Mini proofing chamber (Prática Technipan, Pouso Alegre, Brazil) at 32 °C and 90% relative humidity for 1 h and then baked in a ConvectionLine oven (Venâncio, Venâncio Aires, Brazil) at 155 °C for 35 min. Subsequently, the bread was cooled to room temperature, stored in high-density polyethylene packaging, and kept at room temperature for 24 h.

#### 2.2.9. Dough Volume Increase

The dough volume increase was measured in triplicate by placing 40 g of dough in a 250 mL graduated cylinder and subjecting it to the fermentation process at 32 °C in a BOD RFE38 incubator (Lucadema, São José do Rio Preto, Brazil) [25]. The dough volume increase was recorded every 10 min for 60 min. Using the data of fermentation time versus volume increase, a graph was plotted, and the area under the curve (AUC) was calculated using the OriginPro 8^®^, 2019—Data Analysis and Graphing Software (OriginLab^®^, Northampton, MA, USA).

#### 2.2.10. Technological Aspects of the Bread

The loaves were analyzed for specific volume, instrumental color, and texture of the crumb. The loaves were mechanically sliced in an FPV12 slicer (Venâncio, Venâncio Aires, BRA), and slices with 12 mm thickness were obtained for crumb color and texture analysis.

##### Specific Volume

The specific volume of the loaves was determined using the millet seed displacement method from AACCI (method 10-05.01) [19]. The weight of the loaves was recorded on an S2202 semi-analytical balance (WebLabor, Piracicaba, Brazil), and the volume of millet seeds displaced was measured in a 1000 mL graduated cylinder. The analysis was conducted in triplicate, and the results were expressed in cm^3^·g^−1^ (Equation (3)).

(3) $$Specific\;Volume=\frac{V}{M}$$

where *V* is the volume of displaced millet seeds (cm^3^) and *M* is the weight of the loave (g).

##### Crumb Instrumental Color

The instrumental color of the crumb of the bread was determined using a CM-5 Konica colorimeter (Minolta, Chiyoda, Japan), in the L*, a*, b* color space. Readings were taken with illuminant D65, a 10° viewing angle for the observer, and the RSEX calibration mode [26]. The color difference (ΔE) of the other samples compared to the standard formulation was calculated using Equation (4).

(4) $$∆E=\sqrt{{{(∆L}^{*})}^{2}+{{(∆a}^{*})}^{2}+{{(∆b}^{*})}^{2}}$$

The whiteness index (WI) was calculated according to Equation (5) [27].

(5) $$WI=100-\sqrt{{\left(100-L\right)}^{2}+a^{2}+b^{2}}$$

##### Instrumental Texture

The texture profile analysis was performed using a TA-XT Plus texture analyzer (Micro Systems Stable, Surrey, UK) following AACCI method 74-09.01 [19]. The analysis utilized the P36R probe and the HDP platform. The parameters used were as follows: pre-test speed (1 mm·s^−1^), test speed (1 mm·s^−1^), and post-test speed (10 mm·s^−1^), probe height (30 mm), limiar threshold (0.049 N), and compression distance (40%), for which two slices of 12 mm thickness were overlapped. The analysis of the crumb samples included 10 readings for each test, and the recorded parameters were firmness (N), hardness (N), elasticity (%), cohesiveness (%), gumminess (N), chewiness (N), and resilience (%).

#### 2.2.11. Numerical Optimization of Breadmaking

The optimization of the germination process for bread production was performed following the methodology proposed by Derringer and Suich (1980) [21]. The independent variables were optimized within the studied range. The statistically significant dependent variables were established as minimum values, except for the resilience variable, which was maintained in range. An importance level was assigned to each response (where 1 and 5 represented the lowest and highest levels of importance, respectively).

#### 2.2.12. Statistical Analysis

The results obtained were evaluated using the Response Surface Methodology for regression coefficient calculations and analysis of variance (ANOVA), with a significance level of 10% and a minimum coefficient of determination of 0.80. For numerical optimization, the statistically significant dependent variables that assess the grain’s behavior regarding physiological characteristics (radicle size and seed vigor), nutritional aspects (TSPC and GABA), and biochemical features (action of endogenous enzymes on macromolecules, considering the contents of soluble protein, soluble solids, and reducing sugars) were considered.

## 3. Results and Discussion

### 3.1. Radicle Lenght and Seed Vigor

**Figure 2:**
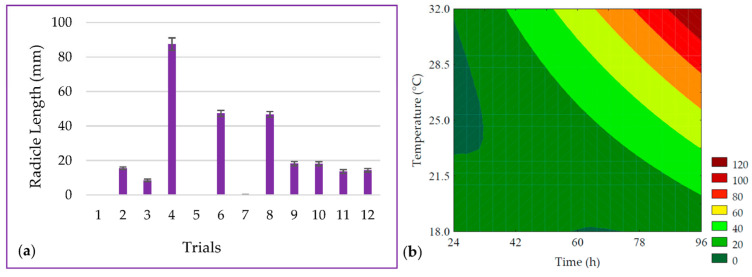
(**a**) Data for the length of the radicle from the seeds and (**b**) contour plot for the radicle length of purple pericarp creole corn under different germination time and temperature conditions according to the Central Composite Design. For trials 1 and 3, no visible radicle was observed, and for trial 7, the beginning of radicle was observed, but measurement with a caliper was not possible. For these samples, a value of zero was adopted for statistical analysis.

The length of the PPCC radicle ranged from 0 to 83.43 mm, as shown in Figure 2a. It was observed that the linear terms of germination time (*β*_1_ = 20.20; *p* < 0.001) and temperature (*β*_2_ = 18.29; *p* < 0.001) had the most significant effect on radicle length. It was possible to develop the predictive mathematical model through ANOVA (R^2^ = 97.63, F_calc_/F_tab (5; 6; 0.10)_ = 15.93, and *p* < 0.001), as presented in Equation (6), as well as the contour plot shown in Figure 2b.

(6) $$Radicle\;Lenght\;\left(mm\right)=16.04\;+\;20.20x_{1}\;+\;18.29x_{2}\;+\;4.89x_{1}^{2}\;+\;4.80x_{2}^{2}\;+\;15.86x_{1}x_{2}$$

where *x*_1_ and *x*_2_ are the encoded levels of the germinatio’s time and temperature, respectively.

**Figure 3:**
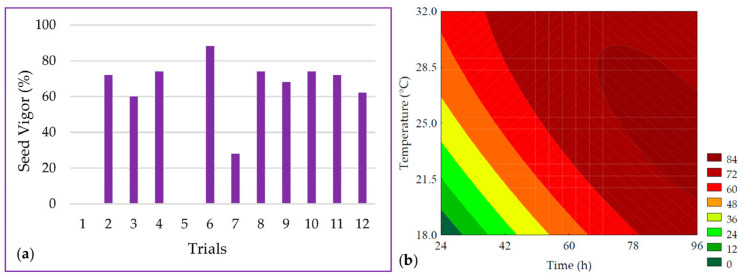
(**a**) Data and (**b**) contour plot for the seed vigor of purple pericarp creole corn under different germination time and temperature conditions according to the Central Composite Design. For trials 1 and 3, no visible radicle was observed, and a value of zero was adopted for statistical analysis.

The seed vigor ranged from 0 to 88% in the experimental design trials, as depicted in Figure 3a. A total of 96.85% of the data was explained by the predictive mathematical model (Equation (7)). The ANOVA showed that the F_calc_/F_tab (5; 6; 0.10)_ ratio was 11.88 with a *p* < 0.001, allowing for the generation of the contour plot presented in Figure 3b. Seed vigor values were maximized by the linear term of time (*β*_1_ = 26.34; *p* < 0.001), while the interaction between time and temperature (*β*_12_ = −14.50; *p* = 0.012) exhibited an antagonistic effect on the response, suggesting that at lower germination temperatures, longer durations would be required.

(7) $$Seed\;Vigor\;\left(\%\right)=68.99+\;26.34x_{1}\;+\;15.90x_{2}-\;11.53x_{1}^{2}-\;8.01x_{2}^{2}-\;14.50x_{1}x_{2}$$

where *x*_1_ and *x*_2_ are the encoded levels of the germination’s time and temperature, respectively.

A seed is considered germinated when it exhibits a visible radicle. However, the germination process begins with the immersion of seeds in water, triggering a gradual reactivation of grain metabolism during the soaking step, accompanied by a series of chemical and biochemical reactions that ultimately lead to the emergence of the radicle [4]. As a result, radicle growth plays a crucial role in determining seed vigor, which serves as one of the primary indicators of seed quality, signifying the potential for a seed to germinate rapidly and uniformly under many conditions [28].

The growth of the radicle is driven by the catabolism of seed macronutrients, including starch, proteins, and lipids, which are hydrolyzed by inherent endogenous enzymes in the germination process. This hydrolysis leads to structural, techno-functional, and quantitative alterations in these macronutrients [29]. Time and temperature are critical factors in the biotechnological process of germination. In this study, it was observed that trials with shorter germination times (trials #1 and #5) did not exhibit a visible radicle but, rather, a small pericarp opening preceding radicle elongation. Consequently, in these trials, visible vigor to the naked eye was absent, underscoring the significance of time in the germination process, which is essential for seedling development and also affects growth and other developmental phases [30].

In addition to time, temperature also holds paramount importance for radicle growth and, subsequently, seed vigor. It deeply influences the enzymatic activity responsible for the breakdown of macronutrients in favor of radicle growth. In this study, it was observed that samples subjected to lower temperatures (trial #7), despite having relatively long germination times (60 h), displayed radicles shorter than 1 mm. This suggests that, while not entirely lacking in vigor, this temperature delayed the seed’s metabolic processes.

### 3.2. Analysis of Germinated and Non-Germinated Corn Flours

The results obtained for TSPC and GABA for germinated and non-germinated (control) PPCC are presented in Table 2.

#### 3.2.1. Total Soluble Phenolic Compounds

**Figure 4:**
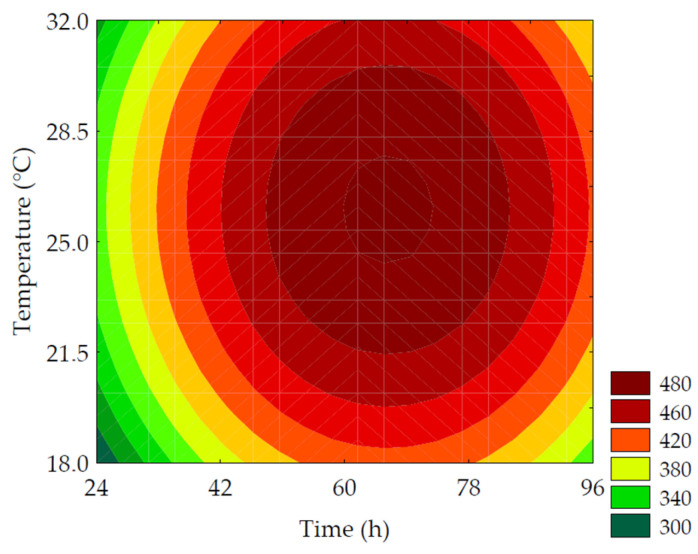
Contour plots for the total soluble phenolic compounds (mg GAE·100 g^−1^ of flour, in dry basis) of purple pericarp creole corn under different germination time and temperature conditions according to the Central Composite Design.

The TSPC content ranged from 342.36 to 497.12 mg GAE·100 g^−1^ (on a dry basis). The linear terms of germination time (*β*_1_ = 23.52; *p* = 0.001) and temperature (*β*_2_ = 11.17; *p* = 0.040) had the most significant impact on TSPC, while the quadratic terms of these variables had a negative influence. The ANOVA (R^2^ = 95.31, F_calc_/F_tab (4; 7; 0.10)_ = 12.02, and *p* < 0.001) allowed for the development of the predictive mathematical model (Equation (8)) and the corresponding contour plot (Figure 4). The highest values of TSPC were obtained with slightly higher germination time and temperature than the central point, resulting in a 1.43-fold increase compared to non-germinated PPCC. The increase in TSPC during germination can be attributed to the release and biosynthesis of these compounds through the degradation of the grain’s cell wall (ester and ether bonds) by endogenous enzymes, such as ligninases and esterases, which release phenolic compounds, specifically hydroxycinnamates, such as ferulic and *p*-coumaric acids, bound to non-starchy polysaccharides [31]. The synthesis and bioconversion of different phenolic compounds have glucose as the original precursor and utilize several important molecular signaling pathways, such as the pentose phosphate pathway, glycolysis, acetate/malonate pathway, and hydrolyzable tannin pathway [6]. The phenylpropanoid pathway is primarily responsible for phenolic compound formation. Phenylalanine ammonia-lyase is a key enzyme in the phenylpropanoid pathway, catalyzing the bioconversion of phenylalanine into cinnamic acid, which is further converted into derivatives, such as coumaric, caffeic, ferulic, and sinapic acids, through a cascade of reactions. The name of the pathway refers to the common phenylpropane (C6-C3) structure found in various phenolic compounds. Through chalcone synthase, a second aromatic ring originating from the acetate pathway combines with the phenylpropane structure, forming the phenylpropane-phenyl (C6-C3-C6) structure, known as chalcone, common to flavonoids [32].

(8) $$TSPC=479.34+\;23.52x_{1}\;+\;11.17x_{2}\;-48.18x_{1}^{2}-\;27.35x_{2}^{2}$$

where *TSPC* is the total soluble phenolic compounds in mg of gallic acid equivalent per 100 g of flour (in dry basis); *x*_1_ and *x*_2_ are the encoded levels of the germination’s time and temperature, respectively.

#### 3.2.2. *γ*-Aminobutyric Acid (GABA)

GABA is a metabolite of interest due to its role in regulating neural activity and is one of the indicators of germination efficiency, originating from the bioconversion of glutamic acid. The data obtained for GABA content ranged from 43.38 ± 0.19 to 61.13 ± 2.69 mg of GABA·100 g^−1^ (on a dry basis) for the trials (Table 2). According to the ANOVA, 98.41% of the data was explained by the predictive mathematical model, with a F_calc_/F_tab (5; 6; 0.10)_ ratio of 23.98 and *p* < 0.001. As shown in Equation (9), the linear term of time (*β*_1_ = 4.72; *p* = 0.002) had a more pronounced effect on increasing GABA values, which is demonstrated by trials #4 and #6 that showed the highest increases in neurotransmitter content (34.35% and 40.82%, respectively, compared to the control). This was followed by the linear term of temperature (*β*_2_ = 3.98; *p* = 0.003).

**Figure 5:**
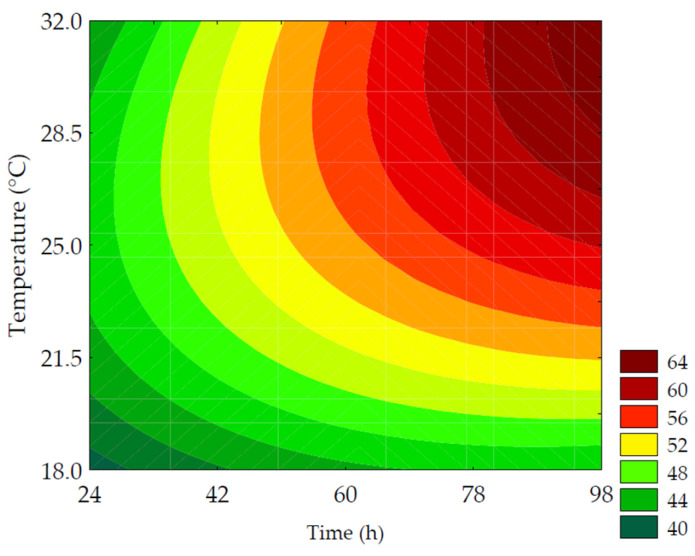
Contour plots for the *γ*-aminobutyric acid in mg of GABA·100 g^−1^ of flour (in dry basis) of purple pericarp creole corn under different germination time and temperature conditions according to the Central Composite Design.

The contour plot (Figure 5) revealed that the highest GABA values were obtained under conditions with longer germination times combined with temperatures between 25 and 30 °C. This behavior is explained by the activity of glutamate decarboxylase (GAD), an endogenous enzyme in plant cells. This enzyme is activated due to abiotic stress induced during germination, leading to the decarboxylation of L-glutamic acid, resulting in the production of CO_2_ and GABA [12,33,34].

(9) $$GABA=53.45+4.72x_{1}+3.98x_{2}-0.98x_{1}^{2}-2.19x_{2}^{2}+2.09x_{1}x_{2}$$

where *GABA* is the *γ*-aminobutyric acid in mg of GABA per 100 g of flour (in dry basis); *x*_1_ and *x*_2_ are the encoded levels of the germination’s time and temperature, respectively.

### 3.3. Analysis of Water-Soluble Extracts

The results obtained for soluble protein, reducing sugars, and soluble solids in the water-soluble extracts from the germinated and non-germinated (control) PPCC are presented in Table 3.

#### 3.3.1. Soluble Protein

**Figure 6:**
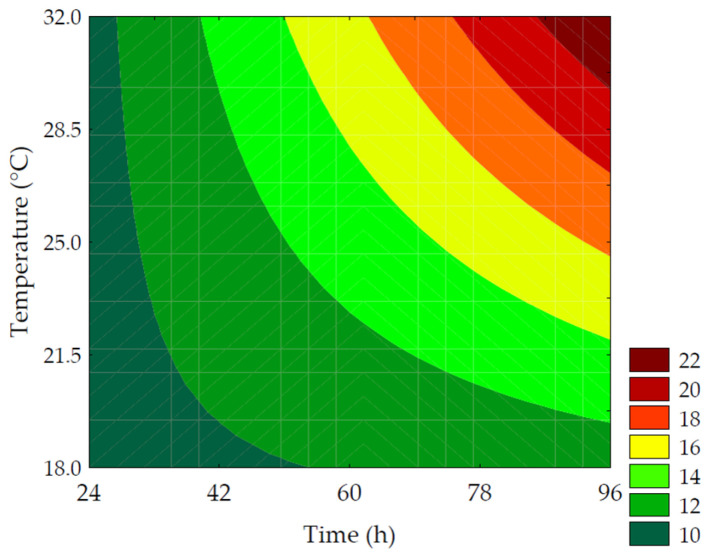
Contour plots for the soluble protein (%) of the water-soluble extracts obtained from the purple pericarp creole corn under different germination time and temperature conditions according to the Central Composite Design.

The content of soluble protein quantified in the water-soluble extracts ranged from 9.31 ± 0.13 to 17.58 ± 1.11% in the experimental design trials. It was observed in Equation (10) that the linear terms of time (*β*_1_ = 2.49; *p* = 0.001) and temperature (*β*_2_ = 1.93; *p* = 0.002), as well as the interaction term between germination time and temperature (*β*_12_ = 1.36; *p* = 0.018), had a positive impact on the responses. The ANOVA explained 95.75% of the results (F_calc_/F_tab (3; 8; 0.10)_ = 20.61, and *p* < 0.001), allowing for the creation of the contour plot (Figure 6). Throughout the germination process, changes occurred in the grain’s biochemical characteristics, resulting in alterations in the profile of reserve proteins that affect their biological functions and enhance their bioavailability. This is supported by the 2.09-fold increase in protein solubility in the water-soluble extract, as observed in trial #6 (96 h of germination at 25 °C) compared to the control, highlighting the significance of germination time in protease activation. Proteolytic enzymes break down the proteins stored in the endosperm to provide nitrogenous compounds for root development [35].

(10) $$Soluble\;Protein\;\left(\%\right)=12.84+\;2.49x_{1}\;+\;1.93x_{2}+1.36x_{1}x_{2}$$

where *x*_1_ and *x*_2_ are the encoded levels of the germination’s time and temperature, respectively.

Plant proteins naturally have low solubility in aqueous environments. In the case of maize, the main proteins are prolamins (also known as zeins), which exhibit low solubility in water due to a higher number of disulfide bonds between cysteine radicals. Therefore, the germination process facilitates the solubilization of these macronutrients into the continuous phase, improving yield and directly affecting the increase in protein digestibility [36]. In this study, it was observed that all trials demonstrated higher protein solubility compared to the control due to the proteolysis that occurs to supply nitrogenous compounds to the grain. This increase in soluble protein content indicates enhanced metabolic activity, leading to the release of free amino acids, peptides, and polypeptides that can later contribute to the biosynthesis of bioactive compounds [37,38]. Additionally, the mashing process employed contributed to maximizing the enzymatic action of proteases from germinated maize, favoring the activity of exopeptidases and endopeptidases [18].

On the other hand, in the case of very prolonged germination processes, a reduction in protein content can be observed due to the catabolism of the embryo in favor of root growth, which was not observed in this study.

#### 3.3.2. Soluble Solids

**Figure 7:**
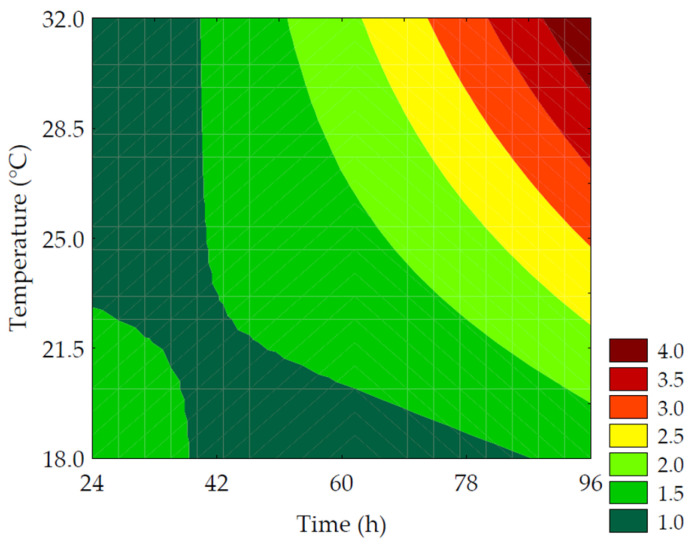
Contour plots for the soluble solids (%) of the water-soluble extracts obtained from the purple pericarp creole corn under different germination time and temperature conditions according to the Central Composite Design.

The enzymatic hydrolysis is promoted by endogenous enzymes, especially amylolytic and proteolytic enzymes acting on reserve macronutrients, such as starch, proteins, and lipids, resulting in low-molecular-weight sugars, amino acids, peptides, and free fatty acids. These compounds modulate respiratory metabolic activities, resulting in an increase in the content of soluble solids available to provide energy for sprout growth and development [39]. Furthermore, enzymes that hydrolyze insoluble fibers favor an increase in soluble fibers during the germination process [3]. The content of soluble solids in the extracts obtained after the mashing process varied between 0.8 and 3.0 °Brix, as shown in Table 3. There was a 275% increase in the soluble solids content when comparing the results of trial #6 to the control. The primary factors influencing the hydrolysis of macromolecules were the linear term of time (*β*_1_ = 0.59; *p* < 0.001) and the interaction of independent variables (*β*_12_ = 0.45; *p* = 0.004). The ANOVA explained 97.50% of the results and enabled the development of a predictive mathematical model (Equation (11)) and the corresponding contour curve (Figure 7), with a F_calc_/F_tab (4; 7; 0.10)_ ratio of 23.08 and *p* < 0.001. Although germination time was more influential than germination temperature, both variables had a positive impact on the content of soluble solids.

(11) $$Soluble\;Solids\;\left(\%\right)=1.34+\;0.59x_{1}\;+\;0.37x_{2}+\;0.19x_{1}^{2}+\;0.45x_{1}x_{2}$$

where *x*_1_ and *x*_2_ are the encoded levels of the germination’s time and temperature, respectively.

Enzymatic hydrolysis is the most important biochemical process in the mashing stage, as it facilitates the solubilization of compounds into the aqueous medium, especially proteins and fermentable carbohydrates, like glucose, maltose, maltotriose, and dextrins. Enzymatic activity is highly temperature-dependent, and as a result, the mashing process contributes to the increase in soluble solids content by promoting the solubilization of compounds through enzymatic action. This leads to the production of a product with higher nutritional and biological value while reducing the loss of compounds after filtration [40].

#### 3.3.3. Reducing Sugar

**Figure 8:**
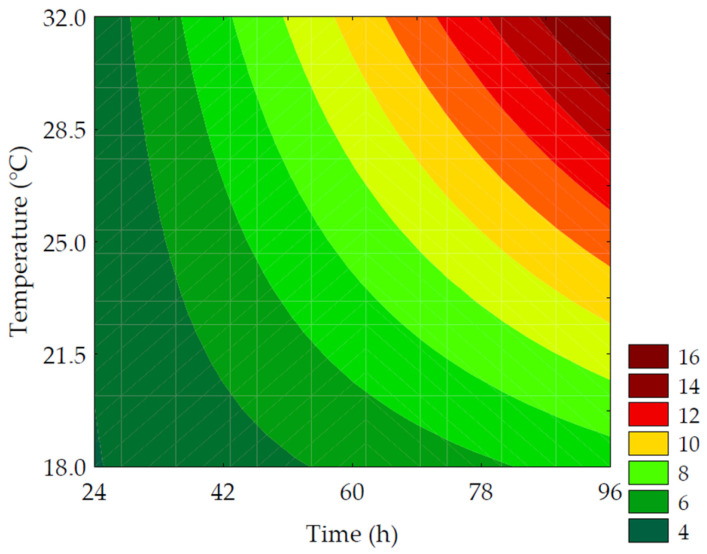
Contour plots for the reducing sugars (g of glucose·100 g^−1^ of the water-soluble extracts) obtained from the purple pericarp creole corn under different germination time and temperature conditions according to the Central Composite Design.

Table 3 presents the results of the analysis of reducing sugars. The total content of reducing sugars in the water-soluble extracts ranged from 3.28 to 10.77 mg of glucose per 100 mL of water-soluble extract. The maximum increase in the levels of reducing sugars compared to non-germinated PPCC (control) was 3.28-times and was achieved with 96 h of germination at 25 °C (trial #6). It was observed that the most significant effect on the total content of reducing groups in carbohydrates was provided by the linear term of germination time (*β*_1_ = 2.24; *p* = 0.003), followed by the linear term of germination temperature (*β*_2_ = 1.47; *p* = 0.008). The ANOVA (R^2^ = 84.88, F_calc_/F_tab (3; 8; 0.10)_ = 5.11, and *p* = 0.001) allowed for the development of a predictive mathematical model and the creation of a contour curve, as presented in Equation (12) and Figure 8, respectively.

(12) $$Reducing\;Sugar=7.28+2.24x_{1}+1.47x_{2}+0.96x_{1}x_{2}$$

where: reducing sugars were expressed as g of glucose·100^−1^ g of extract; *x*_1_ and *x*_2_ are the encoded levels of the germination’s time and temperature, respectively.

The increase in the content of reducing sugars during germination is due to the activation of endogenous amylolytic enzymes, which hydrolyze starch into mono- and disaccharides, primarily responsible for providing energy for root growth [41]. It is observed that in trials with shorter germination times (#1, #3, and #5), there was no significant increase (1.38-fold) in the content of reducing sugars compared to the control trial. This result can be attributed to the low rate of consumption of soluble sugars in the early stages of germination, where the synthesis and activity of amylolytic enzymes are not sufficient for significant starch hydrolysis in the seeds. However, with increasing germination time, the amylolytic enzymes synthesized in the aleurone layer migrate to the endosperm and initiate the hydrolysis of starch granules [42]. Another factor that affects the content of reducing sugars is the germination temperature, as enzymatic activity is reduced at low temperatures. These results can be confirmed by the size of the radicle and seed vigor, as the trial with the lower germination temperature (trial 7) also showed a lower germination rate and reduced root growth.

### 3.4. Numerical Optimization and Validation of Mathematical Models

For the optimization of the germination process to obtain the best condition, the time and temperature parameters were set within the range, with an importance factor of 3. For the statistically significant dependent variables and with predictive mathematical models (*p* < 0.10; F_calc_/F_tab_ > F_tab_ and R^2^ > 0.80), the parameters were maximized, with an importance factor of 5 (Table 4).

The optimal point for the germination process was defined as 85.3 h (85 h and 18 min) at 30.5 °C, with a desirability of 90.42%. As a result, it was observed that the mathematical models were able to predict the findings, as they exhibited a relative deviation of less than 20% [33], thereby validating the mathematical equations.

In this context, germinated PPCC flours can be used in the preparation of water-soluble extracts, as well as in breadmaking as a technological aid to replace commercial flour improvers. Therefore, germinated grains can supply the required enzymes for dough expansion without the necessity of incorporating commercial additives, aligning with consumer expectations for a clean label, featuring a minimalistic and simplified ingredient list, consisting of components easily understood by the public. Additionally, incorporating germinated flours into the formulation can enhance the product’s functional appeal due to the presence of bioactive compounds.

#### Antioxidant Capacity

Considering the validation of the mathematical models, the organic extracts used in the analysis of TSPC for the control and optimum point trials were assessed for their antioxidant capacity using the ORAC method. Purple corn seeds exhibited an antioxidant activity of 264.70 ± 19.82 µmol of Trolox per 100 g of extract. Germination led to an increase in ORAC values to 357.50 ± 35.07 µmol of Trolox per 100 g of extract. According to Paucar-Menacho [43], the high antioxidant activity of purple corn has been associated with its high content of anthocyanins, which are water-soluble compounds. The improvement in antioxidant activity is attributed to the complex biochemical metabolism of conversion and phenolic biosynthesis during the germination process, resulting in the generation of antioxidant phytochemicals, particularly phenolic derivatives [44]. Several studies have demonstrated that phenolic compounds can protect against oxidative stress by activating the antioxidant defense through the positive regulation of antioxidant enzyme expression or by scavenging radicals through the donation of hydrogen and electrons [45].

### 3.5. Dough Volume Increase

**Figure 9:**
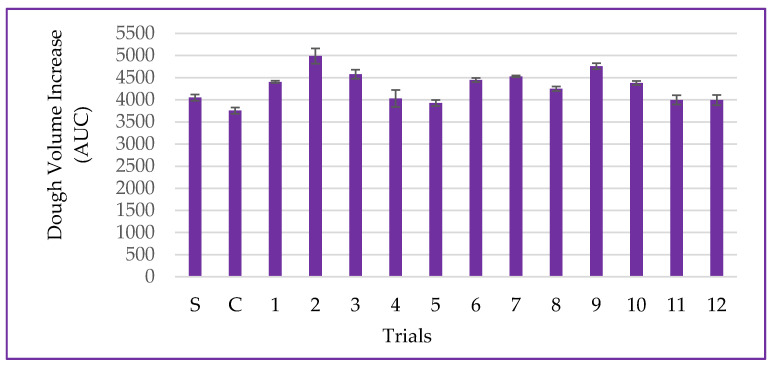
Data for dough volume increase, including the standard (S-only wheat flour), control (C-non-germinated corn flour), and trials 1–12 (germinated corn flour) with 4% corn flour (*w*/*w*) replacing wheat flour.

The dough volume increase is a parameter associated with yeast metabolism during the fermentation process and dough development [25]. In this study, the dough volume increase ranged from 3927 ± 69 to 4988 ± 174 AUC for the DCCR trials, and for the trial without partial wheat flour substitution (standard-S), the obtained value was 4050 ± 70.71 AUC. The use of non-germinated corn flour (control-C) led to a decrease in the dough expansion volume of approximately 7.81%, as shown in Figure 9.

The experimental results for the dough volume increase were explained by the mathematical model in 53.16%, according to the ANOVA (*p* = 0.355), making it impossible to generate contour curves. While the action of amylolytic enzymes may have provided fermentable sugars for yeast metabolism, the action of proteolytic enzymes and the partial dilution of gluten-forming proteins may have weakened the viscoelastic network [24,46].

Furthermore, the stabilization of gas bubbles promoted by the viscoelastic gluten matrix after the hydration and mechanical mixing of ingredients, including starch granules, may have been affected by structural changes in proteins and starch resulting from the actions of endogenous amylolytic and proteolytic enzymes during the germination process, resulting in a decrease in the medium’s viscosity. There are reports that these changes can negatively affect gluten aggregation, primarily because they interfere with water retention capacity, which has deleterious effects on dough viscosity and elasticity [35,47].

### 3.6. Pan Bread Characterization

**Figure 10:**
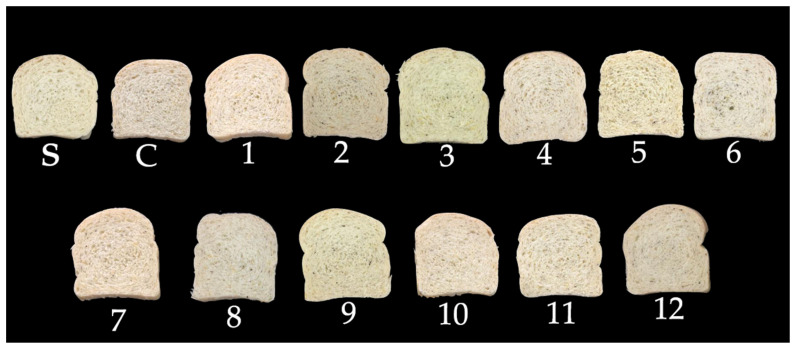
Slices of the loaves, including the standard (S-only wheat flour), control (C-non-germinated corn flour), and trials 1–12 (germinated corn flours) with 4% corn flour (*w*/*w*) replacing wheat flour.

Figure 10 depicts slices of bread obtained in this study for an easier visual understanding of the impact of using PPCC on color characteristics and visual appearance.

#### 3.6.1. Specific Volume

**Figure 11:**
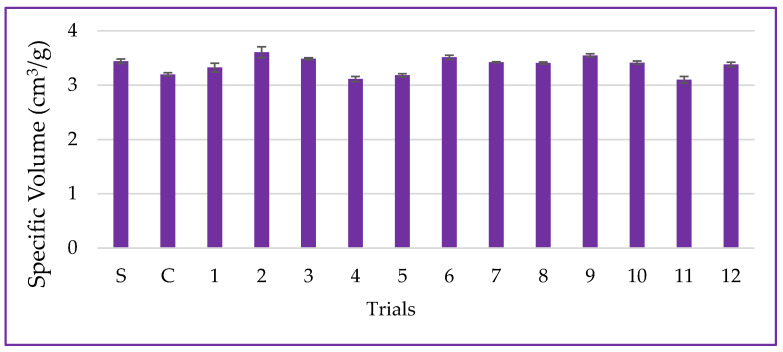
Data for specific volume, including the standard (S-only wheat flour), control (C-non-germinated corn flour), and trials 1–12 (germinated corn flour) with 4% corn flour (*w*/*w*) replacing wheat flour.

Specific volume is the primary quality parameter for bread and is associated with the fermentation of sugars by yeast, which releases CO_2_ to expand the dough [48]. In this study, the specific volume of samples ranged from 3.10 ± 0.06 to 3.61 ± 0.10 cm^3^·g^−1^ for the CCD trials, and it was 3.44 ± 0.04 cm^3^·g^−1^ for the standard sample (S—only wheat flour), as presented in Figure 11. The use of non-germinated PPCC flour (control-C) led to a 7.50% reduction in the specific volume of bread compared to the standard. This result aligns with the dough volume increase and could also be attributed to dietary fibers and non-gluten proteins present in corn flour, inherent to the grain’s chemical structure, promoting a hysteresis barrier and mechanical rupture of the gluten network during the expansion process, ultimately affecting the final product volume [24].

The interaction between germination time and temperature (*β*_12_ = −0.17; *p* = 0.085) had a negative influence on the specific volume of the bread, resulting in a decrease in dough expansion and oven rise. However, the ANOVA analysis indicated that these results can be explained by only 48.95% (*p* = 0.433). The proteases synthesized during the germination process can promote the hydrolysis of dough proteins and the formation of soluble peptides, which may compromise the gluten’s formation and aggregation properties, essential for proper dough development. Consequently, this can affect the quality of the final product, as observed in this study [49]. Therefore, the content of soluble proteins supports this result, as the trial with the highest protein solubility (trial #4) also exhibited a lower specific volume and values below the standard, indicating reduced dough expansion due to proteolytic action. Furthermore, the germination process increased TSPC, as demonstrated previously. These phytochemicals can sequester the sulfhydryl groups (2R—SH) available for the formation of disulfide covalent bonds (R—S—S—R), which may have affected protein aggregation and gluten network formation, as trials with higher TSPC levels also showed lower specific volume values.

#### 3.6.2. Instrumental Color

Color is the first interaction consumers have with food, and, thus, it plays a pivotal role in product acceptance. In the case of bread, the importance of color is primarily associated with the hue of the crumb, which is linked to the ingredients used in its formulation. Bread with a lighter crumb color, characterized by higher luminosity (L*) values, tends to be more favorably received by the public [50].

In this study, L* ranged from 75.25 ± 0.20 to 79.23 ± < 0.01 (Table 5), and it was influenced by the linear term (*β*_2_ = 0.52; *p* = 0.004) and the quadratic term (*β*_22_ = 0.29; *p* = 0.026) of the germination temperature. However, the ANOVA analysis indicated an R^2^ of 58.23% and *p* = 0.274, suggesting that the data were not satisfactorily explained by the mathematical model. The luminosity of the sample with non-germinated PPCC flour (control) decreased, and germinated flours accentuated this reduction compared to the standard, indicating a slight darkening of the crumb. This outcome can be attributed to the inherent pigments, such as anthocyanins and carotenoids, present in the pericarp and endosperm of the grain, respectively. During the germination process and subsequent grinding for particle size reduction (increasing friability), these pigments are exposed more, resulting in darker-crumbed bread [51].

The parameter a* (+a* = red; −a* = green) ranged from 0.08 ± < 0.01 to 0.65 ± 0.02 (Table 5), and this variation was considered not significant (R^2^ = 44.15% and *p* = 0.513). It was observed that the reddish hue (+a*) of the control sample increased compared to the standard due to the presence of anthocyanins inherent to the PPCC pericarp. On the other hand, a* parameter reduction was observed with the addition of germinated flours compared to the control. This result can be attributed to the degradation of anthocyanins during the germination process, which occurs due to grain exposure to moisture, light, and oxygen [4]. Additionally, baking also contributes to the degradation of flavonoids, including anthocyanins.

The b* parameter varied between 19.57 ± 0.10 and 21.16 ± 0.09 (Table 5), and these results were not significantly influenced by the PPCC germination process, as the ANOVA presented R^2^ values of 50.99% and *p* = 0.392. All trials exhibited a reduction in the yellow color (+b*), resulting from the dilution of carotenoids present in wheat flour. This effect was more pronounced in trials #4, #6, and #8 due to the increased release of reducing sugars through enzymatic action, which promoted browning via the Maillard reaction [24].

The instrumental color difference (ΔE) ranged from 1.15 ± 0.40 to 3.00 ± 0.19 (Table 5). The ANOVA was only able to explain 44.42% of the results (*p* = 0.508), making it impossible to generate contour curves and the mathematical model. However, it was observed that the linear and quadratic terms of temperature (*β*_2_ = −0.33; *p* = 0.005; *β*_22_= −0.20; *p* = 0.026) influenced the reduction in color difference compared to the standard. According to Table 5, ΔE was more pronounced in the control sample due to the inherent pigments of PPCC, which were better preserved in the non-germinated sample. This result is supported by the L*, a*, and b* values of the control in comparison to the standard. The trials with the addition of germinated flours showed smaller variations in instrumental color compared to the standard when compared to the control sample. These results can be attributed to the degradation of anthocyanins and carotenoids in the grain due to the process conditions, especially by the germination temperature, which confirms the other instrumental color results of the trials. The instrumental color difference remained below 5 for all trials, indicating that untrained assessors would not be able to distinguish between the samples in terms of color [52]. This result is promising, as significant color differences can lead to product rejection by consumers due to their sensory memory of the traditional product [50].

The WI ranged from 68.00 ± 0.19 to 70.24 ± 0.35 for the experimental trials (Table 5), and the germination process of PPCC did not significantly impact these data, as the ANOVA showed R^2^ values of 63.36% with *p* = 0.200. The linear term of temperature (*β*_2_ = 0.525; *p* = 0.020) contributed to the increase in WI. This result corroborates the difference in instrumental color and brightness of the samples, as the trials with lower L* and ΔE values also exhibited higher WI (trials #4 and #8) and higher process temperatures, which accentuated the degradation of color compounds in both the pericarp and endosperm of the grain. Therefore, the whiteness index of the samples contributed to the reduction in darkening and, consequently, minimized the instrumental color difference, potentially enhancing consumer acceptance of the product.

#### 3.6.3. Instrumental Texture

The texture is one of the key quality parameters for bakery products and is closely related to the specific volume and the product’s alveolar structure. Thus, the texture profile analysis (TPA) correlates instrumental parameters with potential sensory acceptance by consumers, as it mimics the chewing process [24]. The measurements obtained for firmness, hardness, elasticity, cohesiveness, gumminess, chewiness, and resilience are presented in Table 6.

Low values of firmness and hardness are indicative of crumb softness and are more likely to be accepted by consumers. Thus, the release of fermentable sugars is of paramount importance for the production of CO_2_ by yeast during dough fermentation and the subsequent expansion of the alveolar structure, reducing the firmness and hardness parameters of the crumb. These parameters are directly related to the specific volume of the loaf [49].

The instrumental firmness (N) corresponds to the force required for the deformation of 3/5 of the total sample compression, and in this study, it ranged from 4.60 ± 0.27 to 9.73 ± 0.88 N. The linear term of germination time (*β*_1_ = −1.01; *p* = 0.048) contributed to the reduction in crumb firmness, but only 68.66% of the data were explained by the mathematical model (*p* = 0.135). The crumb hardness (N) corresponds to the force required to compress 40% of the sample [25]. In this study, hardness ranged from 6.19 ± 0.40 to 11.04 ± 1.03 N, where the linear term of germination time (*β*_1_ = −0.82; *p* = 0.067) had a significant effect on hardness reduction, but the experimental values were not satisfactorily explained by the mathematical model, with the ANOVA presenting R^2^ values of 62.72% and *p* = 0.209.

The control sample exhibited higher firmness and hardness values compared to the standard due to the low enzymatic activity in the non-germinated matrix and the lower grain friability, which does not favor particle size reduction. This is attributed to the density of the pericarp fibers, which contribute to the mechanical disruption of the mass during the expansion process [51]. These results are supported by the dough volume increase, which was lower than that of the standard sample (Figure 9).

Regarding the other trials, the release of reducing sugars and the radicle length contribute to the reduction in firmness and hardness parameters of the samples (Table 6). During the germination process, there is the synthesis of amylolytic enzymes that hydrolyze starch into lower-molecular-weight sugars to support radicle growth, leading to an increase in the radicle and, consequently, an increase in total reducing sugars in the medium. Therefore, trials with shorter germination times and lower temperatures (trials #1, #5, and #7) showed lower radicle growth and lower total reducing sugar content compared to the other trials, resulting in a lower impact on the reduction in crumb firmness and hardness.

**Figure 12:**
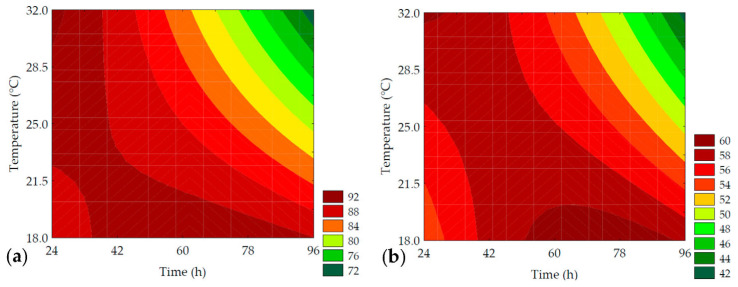
Contour plots for the springiness (%) (**a**) and cohesiveness (%) (**b**) of the bread crumb obtained from the purple pericarp creole corn under different germination time and temperature conditions according to the Central Composite Design.

Springiness (%) and cohesiveness (%) express the ability of the bread crumb to return to its initial state after the application of force in the first compression cycle and the force required to rupture the sample, respectively [25]. Thus, based on Table 6, it was observed that the elasticity of the bread crumb ranged from 78.35 ± 0.83 to 90.45 ± 0.78%, with 91.33% of the results explained by the mathematical model F_calc_/F_tab (4; 7; 0.10)_ = 6.22, and a *p* = 0.001 according to ANOVA. The linear term of time (*β*_1_ = −3.31; *p* = 0.002) and the interaction between the independent variables (*β*_12_ = −2.66; *p* = 0.009) had the most significant effect on the bread crumb’s springiness, as well as the linear term of temperature (*β*_2_ = −2.39; *p* = 0.004) and the quadratic term of time (*β*_11_= −0.99; *p* = 0.061), though to a lesser extent. Cohesiveness ranged from 46.15 ± 0.72 to 59.33 ± 1.12%, with 88.08% of the results supported by ANOVA (F_calc_/F_tab (4; 7; 0.10)_ = 4.37 and *p* = 0.002). The most significant factor in reducing the cohesiveness of the dough was the interaction between germination time and temperature (*β*_12_ = −2.79; *p* = 0.008), followed by the linear term of germination time (*β*_1_ = −2.12; *p* = 0.006). Contour plots, as well as the mathematical models of elasticity and cohesiveness parameters, are presented in Figure 12 and Table 7, respectively.

It was observed that the control sample exhibited lower springiness compared to the standard, indicating that it undergoes more deformation after the first compression cycle. These results can be confirmed by the firmness and hardness results (Table 6), as well as the specific volume of the bread and dough expansion (Figure 9 and Figure 11), which show that the control sample had a denser crumb due to the low enzymatic activity of the non-germinated grain and the presence of pericarp fibers.

The retrogradation of starch is one of the main factors responsible for the aging of bakery products, with texture being the primary affected parameter. In this context, it was observed that the force applied to the food until the point of swallowing (gumminess (N)) was also favored by the incorporation of germinated PPCC flours into the formulation. According to Table 6, gumminess ranged from 3.18 ± 0.25 to 5.90 ± 0.45 N, and 85.78% of the results were explained by the mathematical model (F_calc_/F_tab (3; 8; 0.10)_ = 5.51, and *p* = 0.001), as shown in Table 7. The model indicated that the linear terms of time (*β*_1_= −0.62; *p* = 0.001) and temperature (*β*_2_ = −0.38; *p* = 0.004) of germination contributed to reducing gumminess, while the interaction of variables (*β*_12_ = 0.30; *p* = 0.020) increased it. Thus, the incorporation of germinated corn flours into the formulations increased elasticity and reduced the cohesiveness and gumminess of the crumb. These results can be attributed to the action of maltogenic alpha-amylases that act on the starch cluster structure and reduce amylopectin branched chains, which slows down starch retrogradation in favor of forming a more elastic and less cohesive crumb [53], which may also result in an extended shelf life of the product.

In both cases, the linear parameter of time favored these parameters. Therefore, it can be inferred that maximizing time may be favorable for the synthesis of maltogenic alpha-amylases, promoting starch hydrolysis for radicle growth. These results can also be confirmed by the increase in soluble solids content of the samples, which were maximized by germination time (Table 3), indicating greater enzymatic action. Thus, the action of amylases in releasing fermentable sugars and reducing the size of amylopectin chains is favorable for obtaining desirable texture parameters for the product.

The addition of germinated PPCC flours significantly influences the time required for chewing the product until swallowing (chewiness (N). Chewiness ranged from 2.49 ± 0.20 to 5.17 ± 0.41 N (Table 6), and in this case, it was observed that time (*β*_1_ = −0.67; *p* = 0.011) and temperature (*β*_2_ = −0.43; *p* = 0.034) influenced the reduction in this parameter. On the other hand, the percentage of sample recovery after the first compression cycle (resilience %) was significantly altered with the addition of germinated PPCC flours.

**Figure 13:**
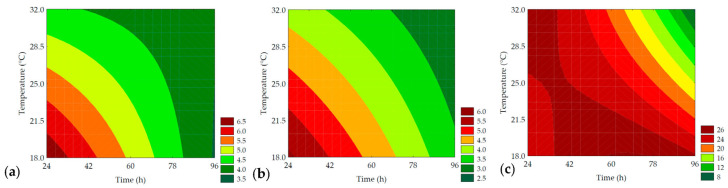
Contour plots for the gumminess (N) (**a**), chewiness (N) (**b**), and resilience (%) (**c**) of the bread crumb obtained from the purple pericarp creole corn under different germination time and temperature conditions according to the Central Composite Design.

Based on Table 6, it was observed that resilience ranged from 13.35 ± 0.31 to 25.54 ± 0.61%, with 97.67% of the results explained by ANOVA (F_calc_/F_tab (4; 7; 0.10)_ = 6.22, and *p* < 0.001), allowing for the generation of the mathematical model (Table 7) and the contour plot (Figure 13). It was observed that the linear term of time (*β*_1_ = −2.79; *p* < 0.001), as well as the interaction term of the independent variables (*β*_12_ = −2.53; *p* = 0.002), was responsible for the negative effect on the response. Similar to elasticity, the crumb’s resilience was also less pronounced in trials with longer germination times and higher temperatures, which can be attributed to the action of proteases (trials #4 and #6) (Table 3). During the germination process, in addition to amylolytic enzymes, proteases are also synthesized, and when applied to bread, they can act on the protein in the dough, weakening the gluten network, adversely affecting the texture parameters of the samples, compacting the crumb and making the final product less elastic and resilient [54].

The germination process should, therefore, be optimized not only for the preparation of water-soluble extracts but also for the production of sandwich bread with satisfactory sensory and instrumental properties. While maximizing germination time and temperature has been beneficial from a nutritional perspective, leading to increased levels of free phenolic compounds, GABA, antioxidant activity, total reducing sugars, and soluble proteins, these parameters need to be fine-tuned for sandwich bread production to prevent excessive proteolytic activity, which can have detrimental effects on both the dough and the final product’s properties.

### 3.7. Numerical Optimization

For the optimization of germination aimed at improving the texture profile of bread, time and temperature parameters were established within the range, with an importance factor of 3. For the statistically significant dependent variables with predictive mathematical models (*p* < 0.10; F_calc_/F_tab_ > F_tab_ and R^2^ > 0.80), the parameters were minimized with an importance factor of 5, except for resilience, which was kept within the range and assigned an importance factor of 3 (Table 8).

Maltogenic *α*-amylases are widely used in baking as an anti-aging strategy to reduce starch retrogradation. In this regard, optimizing the germination process conditions to promote greater synthesis of these enzymes and less proteolytic action is of paramount importance for obtaining quality sensory and technological sandwich bread. Based on Table 8, it was observed that the optimized point for bread differed from what was found for water-soluble extracts (85.30 h at 30.46 °C), mainly due to the proteolytic action, which, in the case of beverages, was maximized to enhance the protein yield of the beverage. While this is beneficial for the preparation of aqueous extracts by solubilizing proteins, it harms gluten network proteins, affecting the volume and texture of the bread, which are the main quality parameters of these products. According to Liu et al. (2023), maltogenic ***α***-amylases are thermostable enzymes, and high temperatures can favor the synthesis of these enzymes through grain metabolism.

## 4. Conclusions

The biotechnological germination process significantly increased the functional compounds of PPCC, such as TSPC, GABA, and antioxidant compounds, which can confer functional effects on the product. The biochemical characteristics of the grain were also positively affected, with an increase in soluble protein, soluble solids, and reducing sugars. The optimized germination condition was 85.3 h at 30.46 °C. The addition of germinated PPCC flours significantly improved the instrumental texture properties of the samples (springiness, cohesiveness, gumminess, and resilience). Color parameters (L*, a*, b*, ΔE, and WI) did not show significant changes. These results indicate the potential for a product that is likely to be well received by consumers. In this context, germinated corn flour has shown promise for obtaining water-soluble extracts and for use in sandwich bread as a substitute for commercial flour improvers. This aligns with consumer expectations for clean labeling and adds value to a grain belonging to the local agrobiodiversity of Couto Magalhães de Minas (Brazil) city.

## Figures and Tables

**Table 1:** Encoded and real levels for the germination process of purple pericarp creole corn germinated under different time and temperature conditions.

Trials	Encoded Levels		Real Levels	
	*x*_1_	*x*_2_	*X*_1_ (h)	*X*_2_ (°C)
1	−1	−1	34	20
2	1	−1	86	20
3	−1	1	34	30
4	1	1	86	30
5	−α	0	24	25
6	α	0	96	25
7	0	−α	60	18
8	0	α	60	32
9	0	0	60	25
10	0	0	60	25
11	0	0	60	25
12	0	0	60	25

where: *x*_1_ and *X*_1_; *x*_2_ and *X*_2_ are the encoded and real levels for germination time and temperature, respectively; α = 1.41.

**Table 2:** Results from the analysis of total soluble phenolic compounds and *γ*-aminobutyric acid in the control and experimental design trials to evaluate the influence of germination time and temperature on purple pericarp creole corn.

Trials	Total Soluble Phenolic Compounds (mg GAE·100 g^−1^ of Flour, d.b.)	*γ*-Aminobutyric Acid (mg·100 g^−1^ of Flour, d.b.)
Control	348.05 ± 31.02	43.41 ± 0.30
1	385.29 ± 46.91	43.38 ± 0.19
2	407.13 ± 43.41	48.56 ± 1.27
3	386.60 ± 37.60	47.56 ± 0.06
4	436.48 ± 45.95	61.13 ± 2.69
5	342.36 ± 49.10	44.89 ± 1.50
6	424.58 ± 25.16	58.32 ± 2.40
7	404.15 ± 29.50	43.89 ± 0.39
8	445.62 ± 41.31	54.52 ± 3.50
9	476.49 ± 63.72	52.21 ± 2.12
10	479.34 ± 73.29	54.97 ± 4.71
11	497.12 ± 80.59	52.48 ± 3.87
12	464.40 ± 69.30	54.12 ± 0.41

Legend: GAE = gallic acid equivalent; d.b. = dry basis.

**Table 3:** Results from the analysis of soluble protein, soluble solids and reducing sugars in the control and experimental design trials to evaluate the influence of germination time and temperature on purple pericarp creole corn.

Trials	Soluble Protein (%)	Soluble Solids (°Brix)	Reducing Sugars (g of Glucose·100^−1^ g of Extract)
Control	8.43 ± 0.11	0.8 ± < 0.01	3.28 ± 0.33
1	9.31 ± 0.13	1.0 ± < 0.01	3.74 ± 0.18
2	11.96 ± 0.34	1.2 ± < 0.01	6.37 ± 0.44
3	10.04 ± 0.08	1.0 ± < 0.01	4.01 ± 0.10
4	17.23 ± 0.48	3.0 ± < 0.01	10.46 ± 0.75
5	9.89 ± 0.10	0.8 ± < 0.01	4.54 ± 0.33
6	17.58 ± 1.11	2.6 ± < 0.01	10.77 ± 1.48
7	10.09 ± 0.21	1.0 ± < 0.01	4.87 ± 0.12
8	16.42 ± 0.16	1.8 ± < 0.01	10.09 ± 0.60
9	13.09 ± 0.04	1.2 ± < 0.01	7.98 ± 0.46
10	11.83 ± 0.28	1.4 ± < 0.01	7.29 ± 0.39
11	12.79 ± 0.22	1.2 ± < 0.01	8.88 ± 0.70
12	13.37 ± 0.01	1.4 ± < 0.01	8.37 ± 0.54

**Table 4:** Results for experimental and predictive values and relative deviation regarding the validation of mathematical models.

**Independent Variables**					
**Parameters**	**Goal**	**Importance**		**Solution**	
			**Coded Value**		**Real Value**
Time (h)	In range	3	0.99		85.3
Temperature (°C)	In range	3	1.10		30.46
**Dependent Variables**					
**Parameter**	**Goal**	**Importance**	**Solution**	**Optimal Point Values**	**Relative Deviation (%)**
Radicle Length (mm)	Maximize	5	83.63	72.39 ± 3.94	15.54
Seed Vigor (%)	Maximize	5	75.87	71.33 ± 0.94	6.36
TSPC (mg GAE·100 g^−1^ of flour, d.b.)	Maximize	5	435.15	395.98 ± 31.02	9.89
GABA (mg·100 g^−1^ of flour, d.b.)	Maximize	5	61.15	54.83 ± 2.07	10.73
Soluble Protein (%)	Maximize	5	18.87	19.24 ± 0.68	1.88
Soluble Solids (%)	Maximize	5	2.99	2.93 ± 0.25	2.01
Reducing Sugars (%, in glucose)	Maximize	5	12.13	14.02 ± 0.33	13.46
Desirability 90.42%					

Legend: TSPC = total soluble phenolic compounds (mg GAE·100 g^−1^ of flour, in dry basis); GABA = *γ*-aminobutyric acid (mg of GABA·100 g^−1^ of flour, in dry basis).

**Table 5:** Data for the instrumental color of the bread crumb, including the standard (S-only wheat flour), control (C-non-germinated corn flour), and trials 1–12 (germinated corn flours) with 4% corn flour (*w*/*w*) replacing wheat flour.

Trials	L*	a*	b*	ΔE	WI
Standard	79.23 ± < 0.01	0.08 ± < 0.01	21.16 ± 0.09	-	70.34 ± 0.06
Control	77.18 ± 0.33	0.59 ± < 0.01	20.33 ± 0.27	1.15 ± 0.40	69.43 ± 0.08
1	75.25 ± 0.20	0.52 ± 0.02	20.27 ± 0.08	3.00 ± 0.19	68.00 ± 0.19
2	75.78 ± 0.02	0.58 ± 0.01	20.40 ± 0.19	2.47 ± 0.05	68.33 ± 0.11
3	76.22 ± 0.21	0.44 ± 0.03	20.39 ± 0.04	2.03 ± 0.21	68.67 ± 0.67
4	77.71 ± 0.30	0.65 ± 0.02	19.71 ± 0.21	1.24 ± 0.15	70.24 ± 0.35
5	76.89 ± 0.98	0.42 ± 0.03	20.01 ± 0.12	1.65 ± 0.74	69.42 ± 0.67
6	75.92 ± 0.31	0.48 ± 0.02	19.61 ± 0.25	2.58 ± 0.26	68.94 ± 0.34
7	76.38 ± 0.16	0.45 ± < 0.01	20.27 ± 0.31	1.92 ± 0.21	68.87 ± 0.21
8	77.28 ± 0.72	0.37 ± 0.02	19.57 ± 0.10	1.62 ± 0.39	70.01 ± 0.59
9	75.99 ± 0.23	0.54 ± < 0.01	20.53 ± 0.05	2.25 ± 0.23	68.40 ± 0.18
10	75.86 ± 0.15	0.34 ± 0.01	20.02 ± 0.23	2.48 ± 0.09	68.64 ± 0.26
11	76.26 ± 0.28	0.31 ± 0.02	19.67 ± 0.18	2.24 ± 0.24	69.17 ± 0.26
12	76.15 ± 0.18	0.37 ± 0.03	20.02 ± 0.29	2.21 ± 0.06	68.86 ± 0.32

Legend: WI = whitening index.

**Table 6:** Data for instrumental texture profile analysis of the bread crumb, including the standard (S-only wheat flour), control (C-non-germinated corn flour), and trials 1–12 (germinated corn flours) with 4% corn flour (*w*/*w*) replacing wheat flour.

Trials	Firmness (N)	Hardness (N)	Springiness (%)	Cohesiveness (%)	Gumminess (N)	Chewiness (N)	Resilience (%)
Standard	7.18 ± 0.39	9.29 ± 0.57	89.83 ± 0.44	56.20 ± 0.84	5.22 ± 0,34	4.69 ± 0.31	24.46 ± 0.77
Control	9.73 ± 0.88	11.04 ± 1.03	87.62 ± 1.38	53.54 ± 1.31	5.90 ± 0.45	5.17 ± 0.41	22.18 ± 0.99
1	8.63 ± 0.50	9.73 ± 0.58	88.29 ± 0.78	56.66 ± 0.70	5.51 ± 0.31	4.87 ± 0.29	24.64 ± 0.57
2	5.45 ± 0.55	7.47 ± 0.58	86.97 ± 0.82	55.45 ± 1.96	4.14 ± 0.32	3.60 ± 0.29	22.55 ± 1.20
3	5.55 ± 0.51	7.45 ± 0.68	90.45 ± 0.78	58.51 ± 0.71	4.36 ± 0.37	3.94 ± 0.32	25.54 ± 0.61
4	7.05 ± 0.52	9.04 ± 0.65	78.50 ± 3.58	46.15 ± 0.72	4.17 ± 0.31	3.27 ± 0.28	13.35 ± 0.31
5	9.13 ± 0.40	10.39 ± 0.45	87.66 ± 0.95	53.67 ± 1.03	5.58 ± 0.30	4.89 ± 0.24	22.46 ± 0.90
6	4.60 ± 0.27	6.19 ± 0.40	78.35 ± 0.83	51.31 ± 1.12	3.18 ± 0.25	2.49 ± 0.20	16.81 ± 0.70
7	7.49 ± 0.52	8.37 ± 0.54	89.54 ± 1.01	59.33 ± 1.12	4.97 ± 0.39	4.45 ± 0.35	26.61 ± 0.90
8	4.90 ± 0.27	6.66 ± 0.38	80.50 ± 0.88	53.91 ± 1.05	3.59 ± 0.20	2.89 ± 0.18	19.15 ± 0.31
9	5.31 ± 0.36	7.19 ± 0.45	86.92 ± 0.84	56.90 ± 1.30	4.09 ± 0.22	3.55 ± 0.21	23.26 ± 0.77
10	6.75 ± 0.38	7.53 ± 0.46	85.67 ± 0.81	56.37 ± 1.66	4.24 ± 0.25	3.63 ± 0.21	22.62 ± 0.66
11	8.17 ± 0.49	9.15 ± 0.59	84.94 ± 0.63	55.32 ± 1.32	5.05 ± 0.26	4.29 ± 0.22	22.39 ± 0.95
12	6.01 ± 0.48	8.01 ± 0.60	86.42 ± 0.70	55.05 ± 0.93	4.41 ± 0.33	3.81 ± 0.28	22.17 ± 0.81

**Table 7:** Mathematical models for the dependent variables for texture parameters with statistical significance (*p* < 0.10).

Dependent Variable	Predictive Mathematical Model	R^2^ (%)	*p*-Value	F_calc_/F_tab_
Springiness (%)	86.01 − 3.31*x*_1_ − 2.39*x*_2_ − 0.99*x*_1_^2^ − 2.66*x*_1_*x*_2_	91.33	0.001	6.22
Cohesiveness (%)	56.12 − 2.12*x*_1_ − 1.89*x*_2_ − 1.86*x*_1_^2^ − 2.79*x*_1_*x*_2_	88.08	0.002	4.37
Gumminess (N)	4.37 − 0.32*x*_1_ − 0.38*x*_2_ + 0.30*x*_1_*x*_2_	85.78	0.001	5.51
Chewiness (N)	3.75 − 0.67*x*_1_ − 0.43*x*_2_ + 0.15*x*_1_*x*_2_	88.84	<0.001	8.24
Resilience (%)	22.77 − 2.79*x*_1_ − 2.36*x*_2_ − 1.47*x*_1_^2^ − 2.53*x*_1_*x*_2_	95.67	<0.001	13.06

Legend: *x*_1_ and *x*_2_ are the encoded levels of the germination’s time and temperature, respectively.

**Table 8:** Results for numerical optimization and predictive values for crumb parameters.

**Independent Variables**				
**Parameters**	**Goal**	**Importance**	**Coded Value**	**Real Value**
Time (h)	In range	3	1.41	96.0
Temperature (°C)	In range	3	0.44	29.34
**Dependent Variables**				
**Parameters**	**Goal**	**Importance**	**Solution**	
Springiness (%)	Minimize	5	76.70	
Cohesiveness (%)	Minimize	5	46.91	
Gumminess (N)	Minimize	5	3.52	
Chewiness (N)	Minimize	5	2.72	
Resilience (%)	Is in range	3	13.35	
	Desirability	92.60%		

## Data Availability

All the data presented in this study are available within the article.
